# Typification and new synonyms of tea (*Camellia sinensis*, Theaceae) and one of its infraspecific taxa

**DOI:** 10.3897/phytokeys.271.173297

**Published:** 2026-03-10

**Authors:** Dongwei Zhao, Shixiong Yang

**Affiliations:** 1 Department of Forestry, School of Forestry, Central South University of Forestry and Technology, Changsha, Hunan 410004, China School of Forestry, Central South University of Forestry and Technology Changsha China; 2 CAS Key Laboratory for Plant Diversity and Biogeography of East Asia, Kunming Institute of Botany, Chinese Academy of Sciences, Kunming, Yunnan 650201, China Kunming Institute of Botany, Chinese Academy of Sciences Kunming China

**Keywords:** Epitypification, lectotypification, neotypification, nomenclature

## Abstract

Tea, *Camellia sinensis*, is a world-widely popular beverage source shrub. Its basionym, *Thea sinensis*, was lectotypified as a vague illustration, which cannot precisely exhibit the diagnostic character states of the species. Here, a Linnaean specimen of *Thea bohea* at LINN, *Linnaeus C. 152*, was designated as the epitype of *T. sinensis* to avoid potential confusion. This specimen was also selected as the neotype of *T. viridis*, a heterotypic synonym of *C. sinensis*. *Camellia sinensis var. niaowangensis*, *T. cantoniensis*, *T. chinensis var. pubescens*, *T. oleosa*, and *T. parvifolia* are treated as new heterotypic synonyms of *C. sinensis var. pubilimba*, one of the infraspecific taxa of tea. *Thea parvifolia* is neotypified, and *T. chinensis var. pubescens* is lectotypified here.

## Introduction

Tea, *Camellia sinensis* (L.) Kuntze (Theaceae), is a globally popular beverage source plant (Zhao 2024). It yields huge economic (Li et al. 2022; Silva and Cooray 2022), healthy (Das et al. 2022), and cultural benefits (Zhou et al. 2021). Tea is native to China (Zhao 2024) and was initially documented by the Chinese people (Fang 1998). The beverage was subsequently taken to other countries and enjoyed by different nations (Jackson 1870; Ukers 1935; Zhang et al. 2018).

Linnaeus (1753) created *Thea* L. for *Thea sinensis* L. The genus was treated as a heterotypic synonym of *Camellia* L. (Sweet 1818) and subsequently, tea received its binomial name as a combination proposed by Kuntze (1881) above. The morphological variations of tea were soon discovered by different plant taxonomists. Since Linnaeus’s *T. bohea* L. (an illegitimate replacement name for *T. sinensis*) and *T. viridis* L. were considered to be conspecific (Zhao et al. 2017), more specific and infraspecific names for the species have been proposed (e.g., Hooker 1847; Pierre 1887; Watt 1907; Kitamura 1950; Chang 1981, 1984; Wang et al. 2011; Le et al. 2020). Some taxonomists argued that the diversity occurred within a single species and the efforts to divide them into different taxa were unnecessary (Seemann 1859; Dyer 1874; Cohen-Stuart 1919). By contrast, others suggested that the variations represented distinguishable infraspecific taxa (Pierre 1887; Watt 1907; Kitamura 1950; Sealy 1958; Hô 1991; Ming 2000; Zhao et al. 2017; Zhao 2024) or even distinct species (Wight 1962; Chang 1998).

Griffith (1854) established *C.* sect. *Thea* (L.) Griff. to include *C. bohea* (L.) Sweet, *C. theifera* Griff., and *C. mastersia* Griff. The natural diversity of *C.* sect. *Thea* has not been adequately described until the works of Chinese taxonomists, such as Zhang (1980), Liang and Zhong (1981), and Chang (1981, 1984), since recent research unveiled that China harbored all taxa of *C.* sect. *Thea* (Zhao 2022, 2024, 2025). With comprehensive collections examined, Ming (1992, 1999, 2000; Ming and Bartholomew 2007) accepted four varieties of tea, including *C. sinensis var. sinensis*, *C. sinensis var. assamica* (Royle ex Hook.) Steenis, *C. sinensis var. dehungensis* (Hung T. Chang & B.H. Chen) T.L. Ming, and *C. sinensis var. pubilimba* Hung T. Chang, largely based on the indumenta of the sepals and ovary, and their geographic distributions. Zhao (2024) added *C. sinensis var. sinensis f. formosensis* (Masam. & Suzuki) Kitam. based on Ming’s (2000) taxonomy of tea and suggested that there were four varieties and one form of *C. sinensis*.

However, several more names of infraspecific taxa of tea, including *C. sinensis var. niaowangensis* Q.H. Chen & H. Peng, *T. cantoniensis* Lour., *T. chinensis var. pubescens* Pierre, *T. oleosa* Lour., *T. parvifolia* Salisb., and *T. viridis* L., have been identified with nomenclatural problems, and they are discussed below.

## Materials and methods

Specimens or their images conserved at herbaria (acronyms following Thiers [2026], continuously updated) A, BM, E, G, GXFI, HGAS, IBK, IBSC, K, KUN, L, LINN, NY, P, PE, SING, SYS, TAI, TCD, and US were examined. Names were typified under Arts. 7–9 of the Madrid Code (Turland et al. 2025, hereafter ICN), and the single correct name for each living taxon was identified under Art. 11.4 of the ICN.

## Taxonomic treatment

### Camellia sinensis

Taxon classificationPlantaeEricalesTheaceae

(L.) Kuntze, Um die Erde: 500. 1881.

A48813A5-558A-5FD2-8F33-DB21FB22B245

**Figure 1.**
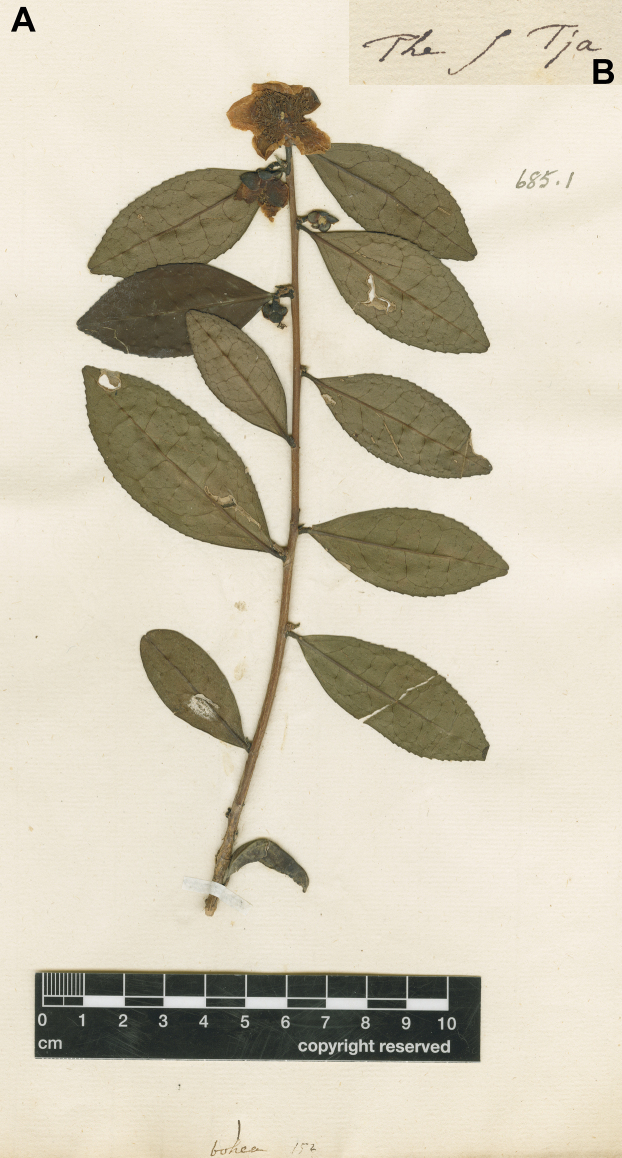
Epitype of *Camellia sinensis* (L.) Kuntze, *Linnaeus C. 152* (LINN). **A**. The obverse; **B**. Records on the reverse. Images were downloaded on the website of LINN (https://linnean.access.preservica.com/uncategorized/IO_fdd28637-3ffd-48a8-bb4a-696bf73fd09b/) and adapted by D.W. Zhao. Reproduced under the terms of the Creative Commons Attribution-Non-Commercial 4.0 International License [CC BY-NC 4.0] (https://creativecommons.org/licenses/by-nc/4.0/).

≡ Thea sinensis L., Sp. Pl. 1: 515. 1753. ≡ Thea bohea L., Sp. Pl., ed. 2. 1: 734. 1762 ≡ Thea grandifolia Salisb., Prodr. Stirp. Chap. Allerton 370. 1796 ≡ Thea chinensis Sims, Bot. Mag. 25: t. 998. 1807 ≡ Camellia thea Link, Enum. Hort. Berol. Alt. 2: 73. 1822 – Lectotype (designated by Bartholomew in Jarvis et al. 1993: 93): “Tsja” in Kaempfer, Amoen. Exot. Fasc.: 606, f. 1–2. 1712 – Epitype (designated here): The s[ive] Tja, *Linnaeus C. 152* (LINN, image! Fig. 1). — An image of the epitype is available at https://linnean.access.preservica.com/uncategorized/IO_fdd28637-3ffd-48a8-bb4a-696bf73fd09b/
= Thea viridis L., Sp. Pl., ed. 2. 1: 735. 1762 – Neotype (designated here): The s[ive] Tja, *Linnaeus C. 152* (LINN, image! Fig. 1).

#### Notes.

Linnaeus (1753) did not designate a type for *T. sinensis*. He subsequently abandoned the name and adopted *T. bohea* (Linnaeus 1762), an illegitimate replacement name for *T. sinensis* (Zhao et al. 2017). After a series of searches of original material in the herbaria listed above, we found that there was a specimen of *T. bohea*, *Linnaeus C. 152*, conserved at herbarium LINN (Fig. 1). This collection bears complete leaves and flowers, which is suitable for acting as the lectotype of *T. sinensis*. Nevertheless, Bartholomew designated an illustration as the lectotype for *T. sinensis* (Jarvis et al. 1993), and his lectotypification must be followed under Art. 9.19 of the ICN (also see Art. 9 Ex. 2 for lectotyping Linnaean names). The illustration (Kaempfer 1712) does not, however, clearly show the diagnostic character states, such as the indumenta of the leaf buds, sepals, and ovaries, for precise identification of *C. sinensis*. Therefore, Linnaean specimen of *T. bohea*, *Linnaeus C. 152* at LINN (Fig. 1), is selected as an epitype of *T. sinensis* to avoid potential confusion (Art. 9.9 of the ICN).

*Thea bohea* and *T. chinensis* are illegitimate replacement names for *T. sinensis* (Zhao et al. 2017). *Thea grandifolia* and *C. thea* are also illegitimate replacement names for *T. sinensis* because *T. bohea* was the sole name cited in their synonymies (Salisbury 1796; Link 1822; Art. 52.2 [e] of the ICN). Linnaeus (1762) described *T. viridis* as “floribus enneapetalis” and recognized *T. bohea* as “floribus hexapetalis”. The number of petals usually varies within a single shrub of tea, so *T. viridis* was recognized as a heterotypic synonym of *C. sinensis* by previous taxonomists (e.g., Sealy 1958; Chang 1984; Ming 2000). The only citation under *T. viridis*, “Hill. exot. t. 22”, is also provided under *T. bohea* (Linnaeus 1762), so it cannot serve as the type material. To avoid further confusion of the names, the epitype of *C. sinensis*, *Linnaeus C. 152* at LINN (Fig. 1), is selected as the neotype of *T. viridis* regardless of the number of petals.

*Camellia sinensis var. sinensis* is native to the subtropical provinces and Guangdong, Guangxi, and Hainan, China. It can be distinguished from other infraspecific taxa by its pubescent leaf buds, acute, shortly attenuate or rounded leaf apex, abaxially glabrous sepals, and pubescent ovaries (Fig. 1; Zhao 2024). As an indigenous popular beverage plant, tea has been collected and planted for at least two millennia in the populous regions of China (Fang 1998). Therefore, it is sometimes difficult to identify a living specimen of tea in the forest as being genuinely wild or having escaped from cultivation (Zhao 2022, 2024).

### Camellia sinensis var. pubilimba

Taxon classificationPlantaeEricalesTheaceae

Hung T. Chang, Acta Sci. Nat. Univ. Sunyatseni 20(1): 98. 1981.

631F278E-5A98-51D2-BDAE-461ACED987E7

= Thea cantoniensis Lour., Fl. Cochinch. 1: 339. 1790, **syn. nov**. – Holotype: *Loureiro s.n*. (P00150892, image!). — An image of the holotype is available at https://science.mnhn.fr/institution/mnhn/collection/p/item/p00150892
= Thea oleosa Lour., Fl. Cochinch. 1: 339. 1790, **syn. nov**. – Holotype: *Loureiro s.n*. (P00150891, image!). — An image of the holotype is available at https://science.mnhn.fr/institution/mnhn/collection/p/item/p00150891
= Thea parvifolia Salisb., Prodr. Stirp. Chap. Allerton 370. 1796 ≡ Camellia sinensis f. parvifolia (Salisb.) Sealy, Rev. Gen. Camellia 116. 1958, **syn. nov**. – Neotype (designated here): *Loureiro s.n*. (P00150891, image!).
= Thea chinensis var. *pubescens* Pierre, Fl. Forest. Cochinch. t. 114 B. 1887, **syn. nov**. – Lectotype (designated here): Pierre, Fl. Forest. Cochinch. t. 114 B. 1887.
var. niaowangensis = Camellia sinensis var. niaowangensis Q.H. Chen & H. Peng, Seed 30(1): 65. 2011, **syn. nov**. – Holotype: China. Guizhou: Guiding, Pingfa, 1400 m, 5 November 2007, *J.H. Wang 07001* (HGAS!).

#### Holotype.

China • Guangxi: Lingyun, Guangxi Forestry Institute 4209 (GXFI!).

#### Notes.

Abaxial sepal indumentum of tea plants is a naturally variable character. Chang (1981) proposed a name, *C. sinensis var. pubilimba*, for the tea plant bearing an abaxially pubescent sepal. However, the earliest names for this kind of plant are *T. cantoniensis* and *T. oleosa*, which were described in 1790, and their types are conserved at herbarium P (also see Sealy 1958). Based on molecular phylogenetic analyses (Shen et al. 2025; Zhao 2025), samples of *C. sinensis var. pubilimba* cannot form a clade, and they are nested in the broad concept of tea, so it should be treated as an infraspecific taxon of tea rather than an independent species. Therefore, *T. cantoniensis* and *T. oleosa* do not bear priority at the infraspecific rank under Arts. 11.2 and 11.4 of the ICN.

Sealy (1958) suggested that *C. sinensis* f. parvifolia bore an abaxially hairy sepal. Since no type was indicated in the protologue of *T. parvifolia* (Salisbury 1796), the holotype of *T. oleosa* (P00150891) is selected as the neotype of *T. parvifolia*. Sealy’s *C. sinensis* f. parvifolia may be the earliest infraspecific name for the plant based on the literature available. However, it is better to treat the tea plant bearing an abaxially hairy sepal as a variety of *C. sinensis* because this variation occurs in the natural distribution areas of both tea and Assam tea (*C. sinensis var. assamica*). Additionally, Chang’s (1981) *C. sinensis var. pubilimba* has been used so widely (e.g., Chang and Bartholomew 1984; Ming 2000; Ming and Bartholomew 2007; Meng et al. 2018; Nguyen et al. 2022; Zhao 2024; Kong et al. 2025) that sustaining its nomenclatural stability will be very helpful (see Art. 14.2 of ICN). Therefore, the plant remains recognized as a variety of tea, and the name, *C. sinensis var. pubilimba*, retains the priority at the varietal rank (Art. 11.4 of the ICN) and accordingly, serves as the single correct name for the taxon.

*Thea chinensis* var. *pubescens* bore an abaxially hairy sepal according to its illustration (Pierre 1887), which is selected as the lectotype of the taxon. *Thea cantoniensis*, *T. chinensis var. pubescens*, *T. oleosa*, and *T. parvifolia* are all treated as the earlier heterotypic synonyms of *C. sinensis var. pubilimba*. A recently published name, *C. sinensis var. niaowangensis* (Wang et al. 2011), is also treated as a heterotypic synonym of *C. sinensis var. pubilimba* because it bears the common diagnostic character state of the sepals.

## Supplementary Material

XML Treatment for Camellia sinensis

XML Treatment for Camellia sinensis var. pubilimba

## Additional information

### Use of AI

No use of AI was reported.

### Author ORCIDs

Dongwei Zhao https://orcid.org/0000-0002-7761-7127

Shixiong Yang https://orcid.org/0000-0001-8472-1275

### Data availability

All of the data that support the findings of this study are available in the main text.

## References

[B1] Chang HT (1981) *Thea*—A section of beveragial tea-trees of the genus *Camellia*. Acta Scientiarum Naturalium Universitatis Sunyatseni 20(1): 87–99. http://xuebao.sysu.edu.cn/Jweb_zrb/CN/Y1981/V20/I1/89

[B2] Chang HT (1984) A revision on the tea resource plants. Acta Scientiarum Naturalium Universitatis Sunyatseni 23(1): 1–12. http://xuebao.sysu.edu.cn/Jweb_zrb/CN/Y1984/V23/I1/3

[B3] Chang HT (1998) Theaceae (1) Theoideae 1. *Camellia*. In: Delectis Florae Reipublicae Popularis Sinicae Agendae Academiae Sinicae Edita (Eds) Flora Reipublicae Popularis Sinicae (Vol. 49[3]). Science Press, Beijing, 3–195.

[B4] Chang HT, Bartholomew B (1984) Camellias. Timber Press, London, 211 pp.

[B5] Cohen-Stuart CP (1919) A basis for tea selection. Bulletin du Jardin Botanique de Buitenzorg 1(4): 193–320.

[B6] Das C, Banerjee A, Saha M, Chatterjee S (2022) A review of the health benefits of tea: Implications of the biochemical properties of the bioactive constituents. Current Research in Nutrition and Food Science 10: 458–475. 10.12944/crnfsj.10.2.5

[B7] Dyer WTT (1874) Ternstroemiaceae. In: Hooker JD (Ed.) The Flora of British India (Vol. 1). L. Reeve & Co., London, 279–294. 10.5962/bhl.title.678

[B8] Fang J (1998) No tea before the Warring States period. Agricultural History of China 17(2): 6–14, 39.

[B9] Griffith W (1854) Notulae ad plantas Asiaticas, part 4. Printed by Charles A. Serrao, Calcutta, 764 pp. 10.5962/bhl.title.70352

[B10] Hô PH (1991) Câycỏ Việtnam (Vol. 1). Mekong Printing, Santa Ana.

[B11] Hooker WJ (1847) Kew gardens; or a popular guide to the Royal Botanic Gardens of Kew. Printed for Longman, Brown, Green, and Longmans, London, 56 pp. 10.1017/cbo9781107324954

[B12] Jackson JR (1870) Tea. Nature 2: 215–217. 10.1038/002215a0

[B13] Jarvis CE, Barrie FR, Allan DM, Reveal JL (1993) A list of Linnaean generic names and their types. Regnum Vegetabile 127: 1–100.

[B14] Kaempfer E (1712) Amoenitatum exoticarum politico-physico-medicarum (Fasciculi V). Typis & Impensis Henrici Wilhelmi Meyeri, Aulae Lippiacae Typographi, Lemgoviae.

[B15] Kitamura S (1950) On tea and camellias. Acta Phytotaxonomica et Geobotanica 14(2): 56–63.

[B16] Kong WL, Kong XR, Xia ZQ, Li XF, Wang F, Shan RY, Chen ZH, You XM, Zhao YY, Hu YP, Zheng SQ, Zhong ST, Zhang SC, Zhang YB, Fang KX, Wang YH, Liu H, Zhang YZ, Li XL, Wu HL, Chen GB, Zhang XT, Chen CS (2025) Genomic analysis of 1,325 *Camellia* accessions sheds light on agronomic and metabolic traits for tea plant improvement. Nature Genetics 57: 997–1007. 10.1038/s41588-025-02135-z PMC11985346 40097782

[B17] Kuntze CEO (1881) Um die Erde. Paul Frohberg, Leipzig.

[B18] Le QU, Nguyen DL, Lay LH (2020) *Camellia sinensis var. dulcamara* (*Camellia*, Theaceae), a new var. and subvar. recorded for sect. *Thea* from Northern Vietnam. Journal on New Biological Reports 9(1): 44–49.

[B19] Li Z, Liu D, Huo ZH, Chen FQ (2022) Analysis on the competitiveness and complementarity of tea trade between China and RCEP members. Journal of Tea Science 42(5): 740–752. https://www.tea-science.com/CN/Y2022/V42/I5/740

[B20] Liang SY, Zhong YC (1981) A new species of Theaceae from China. Acta Scientiarum Naturalium Universitatis Sunyatseni 20(3): 118–119. http://xuebao.sysu.edu.cn/Jweb_zrb/CN/Y1981/V20/I3/120

[B21] Link JHF (1822) Enumeratio Plantarum Horti Regii Berolinensis Altera (Part 2). Apudg. Reimer, Berolini. 10.5962/bhl.title.66

[B22] Linnaeus C (1753) Species plantarum. Impensis Laurentii Salvii, Holmiae. 10.5962/bhl.title.669

[B23] Linnaeus C (1762) Species plantarum, ed. 2. Impensis direct. Laurentii Salvii, Holmiae. 10.5962/bhl.title.11179

[B24] Meng XH, Zhu HT, Yan H, Wang D, Yang CR, Zhang YJ (2018) C-8 N-Ethyl-2-pyrrolidinone-substituted flavan-3-ols from the leaves of *Camellia sinensis var. pubilimba*. Journal of Agricultural and Food Chemistry 66: 7150–7155. 10.1021/acs.jafc.8b02066 29889511

[B25] Ming TL (1992) A revision of *Camellia* sect. *Thea*. Acta Botanica Yunnanica 14(2): 115–132.

[B26] Ming TL (1999) A systematic synopsis of the genus *Camellia*. Acta Botanica Yunnanica 21(2): 149–159.

[B27] Ming TL (2000) Monograph of the genus *Camellia*. Yunnan Science and Technology Press, Kunming, 352 pp.

[B28] Ming TL, Bartholomew B (2007) Theaceae. In: Wu ZY, Raven PH, Hong DY (Eds) Flora of China (Vol. 12). Hippocastanaceae through Theaceae. Science Press, Beijing / Missouri Botanical Garden Press, St. Louis, 366–478.

[B29] Nguyen TT, Luong VD, Le NHN, Quang CT, Yang SX (2022) Taxonomic notes of three tea-plants (*Camellia* sect. *Thea*) recently described in Vietnam. Beverage Plant Research 2: 21. 10.48130/BPR-2022-0021

[B30] Pierre L (1887) Flore forestière de la Cochinchine (Fasc. 8). Octave Doin, Paris. 10.5962/bhl.title.61558

[B31] Salisbury RA (1796) Prodromus Stirpium in Horto ad Chapel Allerton Vigentium. Londini. 10.5962/bhl.title.427

[B32] Sealy JR (1958) A Revision of the Genus *Camellia*. The Royal Horticultural Society, London, 239 pp.

[B33] Seemann B (1859) Synopsis of the genera *Camellia* and *Thea*. Transactions of the Linnean Society of London 22(4): 337–352. 10.1111/j.1096-3642.1856.tb00103.x

[B34] Shen ZF, Feng YJ, Möller M, Burgess KS, Qin HT, Yang JB, Mo ZQ, Li HT, Li DZ, Gao LM (2025) Genomic DNA barcodes provide novel insights into species delimitation in the complex *Camellia* sect. *Thea* (Theaceae). BMC Plant Biology 25: 570. 10.1186/s12870-025-06612-9 PMC12044775 40307692

[B35] Silva MWA, Cooray NS (2022) The export performance of the Sri Lankan tea: An econometric analysis. International Journal of Research and Innovation in Social Science 6: 224–227.

[B36] Sweet R (1818) Hortus Suburbanus Londinensis. Printed for James Ridgway, London, 242 pp. 10.5962/bhl.title.109914

[B37] Thiers BM (2026) Index Herbariorum. http://sweetgum.nybg.org/science/ih/ [accessed 12 February 2026]

[B38] Turland NJ, Wiersema JH, Barrie FR, Gandhi KN, Gravendyck J, Greuter W, Hawksworth DL, Herendeen PS, Klopper RR, Knapp S, Kusber WH, Li DZ, May TW, Monro AM, Prado J, Price MJ, Smith GF, Señoret JCZ (2025) International Code of Nomenclature for algae, fungi, and plants (Madrid Code) accepted by the twentieth International Botanical Congress, Madrid, Spain, July 2024. Regnum Vegetabile 162. The University of Chicago Press, Chicago and London, 303 pp. 10.7208/chicago/9780226839479.001.0001

[B39] Ukers WH (1935) All about tea. The Tea and Coffee Trade Journal Company, New York.

[B40] Wang JH, Chen JH, Lin CH (2011) A new variety of *Camellia* (Theaceae) in Guizhou-mist Hyson. Seed 30(1): 65–66.

[B41] Watt G (1907) Tea and the tea plant. Journal of the Royal Horticultural Society 32: 64–96.

[B42] Wight W (1962) Tea classification revised. Current Science 31: 298–299.

[B43] Zhang FC (1980) Two new species of *Camellia* from Yunnan. Acta Botanica Yunnanica 2(3): 341–344.

[B44] Zhang WJ, Rong J, Wei CL, Gao LM, Chen JK (2018) Domestication origin and spread of cultivated tea plants. Biodiversity Science 26(4): 357–372. 10.17520/biods.2018006

[B45] Zhao DW (2022) Nomenclature, typification, and natural distribution of *Camellia sinensis var. assamica* (Theaceae). Journal of Tea Science 42(4): 491–499. https://www.tea-science.com/CN/Y2022/V42/I4/491

[B46] Zhao DW (2024) Botany and taxonomy of tea (*Camellia sinensis*, Theaceae) and its relatives. In: Chen L, Chen JD (Eds) The Tea Plant Genome. Concepts and Strategies in Plant Sciences. Springer, Singapore, 13–37. 10.1007/978-981-97-0680-8_2

[B47] Zhao DW (2025) *Camellia yangii* (Theaceae), a new species of tea plants (*Camellia* section *Thea*). PhytoKeys 257: 247–256. 10.3897/phytokeys.257.152000 PMC12239013 40636267

[B48] Zhao DW, Parnell JAN, Hodkinson TR (2017) Names of Assam tea: Their priority, typification and nomenclatural notes. Taxon 66: 1447–1455. 10.12705/666.11

[B49] Zhou ZX, Xue C, Ruan HG (2021) An analysis of the spiritual core and value of the Chinese tea culture: A case study of etiquette, customs, ceremonies and traditions of Chinese tea. Journal of Tea Science 41(2): 272–284. https://www.tea-science.com/CN/Y2021/V41/I2/272

